# A statistic approach to electronic moulding versus traditional plaster moulding

**DOI:** 10.1186/1748-7161-7-S1-O42

**Published:** 2012-01-27

**Authors:** JC De Mauroy, F Barral, C Lecante

**Affiliations:** 1 Clinique du Parc, Lyon, France; 2 Groupe Lecante, Lyon, France

## Background

Electronic moulding tends to gradually replace the plaster moulding. Is it as effective?

## Materials and methods

The angular correction of 166 plaster moulding scoliosis was compared with the correction of 117 electronic moulding scoliosis. Both mouldings were made by the same physician. The electronic moulding has been produced using the full 3D system ORTEN. The curves have been grouped into thoracic (n=127), thoracolumbar(n=65) and lumbar(n=206).

## Results

1) The average initial angle is: 28,19 (+-9,21) for thoracic, 28,11 (+-9,34) for thoraco-lumbar and 25,86 (+-7,04) for lumbar curves.

2) The angular reducibility is 54 % in braces for the thoracic curvatures, 69 % for the thoraco-lumbar curvatures and of 73 % for the lumbar curvatures, which corresponds to the usual results of the Lyon management. If we select curves of 30° and more the reducibility is respectively: 48,5 % for thoracic, 67 % for thoraco-lumbar and lumbar curves.

3) The reducibility in brace is better for the group of the electronic moulding than for the group in plaster cast. For all cases, the improvement of reducibility is 3,63° for thoracic, 3,02 for thoraco-lumbar and 2,14° for lumbar curves. This improvement is better if we select the initial curves of 30° and more: respectively 5,44° for thoracic, 4,75° for thoraco-lumbar and 3,89° for lumbar curves. This difference is however not statistically significant.

## Conclusions

Such results are in favour of the electronic moulding, which remains however delicate and require a precise position of the patient during the surface topography and well trained orthotic technicians.

